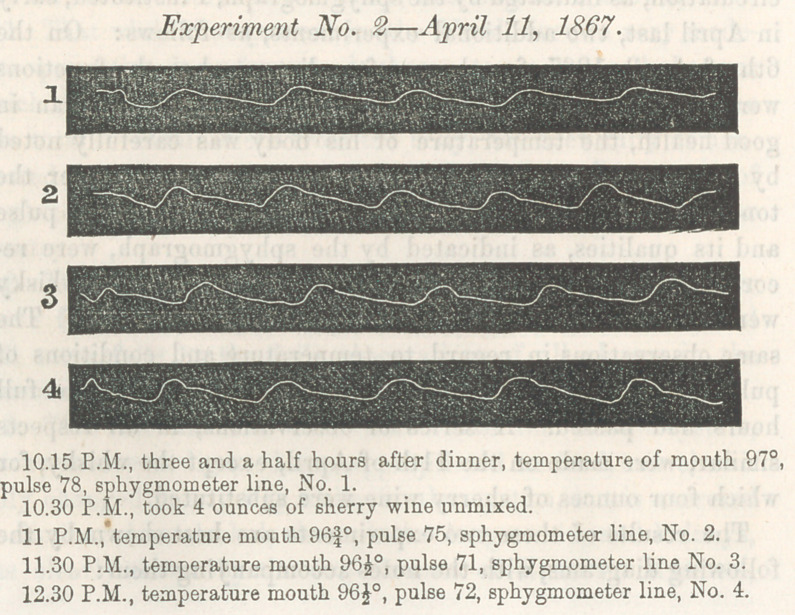# Experimental Inquiries Concerning the Physiological Effects of Alcoholic Drinks on Man

**Published:** 1867-09

**Authors:** N. S. Davis

**Affiliations:** Professor Practical and Clinical Medicine, Chicago Medical College


ARTICLE XXXIX.

### EXPERIMENTAL INQUIRIES CONCERNING THE PHYSIOLOGICAL EFFECTS OF ALCOHOLIC DRINKS ON MAN.

By N. S. DAVIS, M.D., Professor Practical and Clinical Medicine, Chicago Medical College.

Read to the Illinois State Medical Society, June, 1867.

To investigate patiently and thoroughly the modus operandi and effects of such agents, as by their general use both by the profession and the community, are exerting important influences on human health, life, and happiness, is one of the most imperative duties incumbent on the physician. That alcoholic drinks, both fermented and distilled, are so used, and that they are daily exerting an immense influence over human life and happiness is universally acknowledged. Hence we ask your candid and patient attention to the following results of investigations concerning their effects on the human system.

That alcohol, when drank in the form of beer, wine, whisky, or brandy, is rapidly absorbed and carried with the blood into all the structures of the body, has long been known.

1. That while thus present in the blood, even in very moderate quantities, it so far retards those atomic and cell changes which constitute nutrition and disintegration, as to diminish the sum total of eliminations from the body, has been fully demonstrated by the experiments of Drs. PROUT and CHAMBERS, of England, BOECKER, of Germany, HAMMOND and others in our country.

2. That its presence diminishes and disturbs innervation or nerve force, is pretty clearly established by CHAMBERS, and still more fully by the daily observations of all classes in the community.

3. That the alcohol, when taken into the human system, neither becomes digested nor appropriated as food, nor chemically changed in any way while in the system, but is excreted or eliminated as alcohol, through the lungs, skin, and kidneys, was clearly shown by the experiments of RUDOLPH MESSING, in 1854, and fully confirmed by those of LALLEMAND, PERRIN, and DUROY, in 1860.

While the foregoing investigations are sufficient to establish the fact, that alcohol acts simply as a foreign body in the human system, and by its presence retarding the play of vital affinities to such an extent as to materially impair the great functions of tissue change, elimination, and innervation; they left its effects on the circulation and colorification undetermined.

To supply this deficiciency I instituted a series of experiments in 1850, the results of which were communicated to the American Medical Association, at its meeting in Charleston, in May, 1851, and published in the North-western *Medical and Surgical Journal* for that year. These experiments showed that the presence of only a few ounces of either fermented or distilled drinks, in the human system was sufficient to produce a positive diminution of temperature. Two years preceding, namely, in 1848, M. M. DUMEREIL and DUMARQUAY, in making some experiments on intoxicated *dogs*, found their temperature uniformly reduced. For the purpose of verifying still further these results, and adding some coincident observations on the circulation, as indicated by the sphygmograph, I instituted, early in April last, two additional experiments, as follows: On the 6th of April, 1867, four hours after dinner, when the functions were supposed to be undisturbed by digestion, and the man in good health, the temperature of his body was carefully noted by a delicately graduated thermometer inserted under the tongue, with the lips closed around it; the rate of the pulse and its qualities, as indicated by the sphygmograph, were recorded at the same time. Four ounces of bourbon whisky were then administered, diluted with sweetened water. The same observations in regard to temperature and conditions of pulse were made and recorded every half hour, until two full hours had passed. A series of observations, in all respects similar, were made on the 11th of April, except the whisky, for which four ounces of sherry wine were substituted.

The results of these two experiments are best shown by the following diagrams, with the notes accompanying them:

### *Experiment No. 1—April 6, 1867.*

1

2

3

4

5

10.20 P.M., four hours after dinner, temperature of mouth $98\frac{1}{4}$°, pulse 83, sphygmometer line, No. 1.

10.30 P.M., took 4 ounces of Bourbon whisky, with sweetened water.

11 P.M., temperature mouth $97\frac{3}{4}$°, pulse 85, sphygmometer line, No. 2.

11.30 P.M., temperature mouth $97\frac{1}{2}$°, pulse 89, sphygmometer line, No. 3.

12 P.M., temperature mouth $97\frac{1}{2}$, pulse 88, sphygmometer line, No. 4.

12.30 A.M., temperature mouth $97\frac{1}{2}$°, pulse 85, sphygmometer line, No. 5.

### *Experiment No. 2—April 11, 1867.*

1

2

3

4

10.15 P.M., three and a half hours after dinner, temperature of mouth 97°, pulse 78, sphygmometer line, No. 1.

10.30 P.M., took 4 ounces of sherry wine unmixed.

11 P.M., temperature mouth $96\frac{3}{4}$°, pulse 75, sphygmometer line, No. 2.

11.30 P.M., temperature mouth $96\frac{1}{2}$°, pulse 71, sphygmometer line No. 3.

12.30 P.M., temperature mouth $96\frac{1}{2}$°, pulse 72, sphygmometer line, No. 4.

It will be seen that under the influence of the whisky the temperature diminished $\frac{3}{4}$ of a degree in one hour; while under the influence of the same quantity of wine it diminished $\frac{1}{2}$ a degree in the same length of time. Under the influence of whisky the rate of pulsations fluctuated, increasing during the first hour from 83 to 89, and decreasing during the second hour from 89 to 85 per minute. Under the influence of the wine the rate steadily decreased from 78 to 71 or 72. The qualities of the pulse, as indicated by the sphygmograph, are the same in kind, differing only in degree in the two experiments. An inspection of the cuts and accompanying notes will give a much more perfect idea of these representations than any description that can be given in words. It will be seen that each pulse expands the artery to a greater extent and more suddenly than before the alcoholic liquid was taken, and that the commencement of the contraction equally more sudden, while the whole line becomes more wavy or irregular; thereby much resembling the *pulse lines* when the arterial coats are weakened by fatty degeneration; or in such diseases as are accompanied by enfeebled capillary circulation, like typhus and typhoid fevers. Although the inferences to be drawn from the sphygmographic pulse lines as connected with different physiological and pathological conditions of the human system are yet imperfectly understood, yet so far as observations have been made, the lines in the accompanying diagram indicate retarded movement of blood in the capillaries, and consequent increased suddenness of expansion and contraction of the arterial trunks by the impulse of the heart. If this inference be correct, then we may sum up the results of all the varied and ingenious experiments in reference to the effects of alcohol on the human system, many of which have been performed both in this country and Europe, in the two following propositions:

*First.* Its presence in the blood directly interferes with the normal play of vital affinities and cell action in such a manner as to diminish the rapidity of nutrition and disintegration, and consequently to diminish the dependent functions of elimination, calorification, and innervation; thereby making alcohol a positive organic sedative, instead of a diffusable stimulant, as is popularly supposed both in and out of the profession.

*Second.* That the alcohol itself acts in the system exclusively as a foreign substance incapable of assimilation or decomposition by the vital functions, and is ultimately excreted or eliminated without chemical change.

The important bearing of these conclusions on the therapeutic and hygienic uses of alcoholic drinks, must be obvious to all, and especially demand the careful attention of every member of our profession.

## Figures and Tables

**Figure f1:**
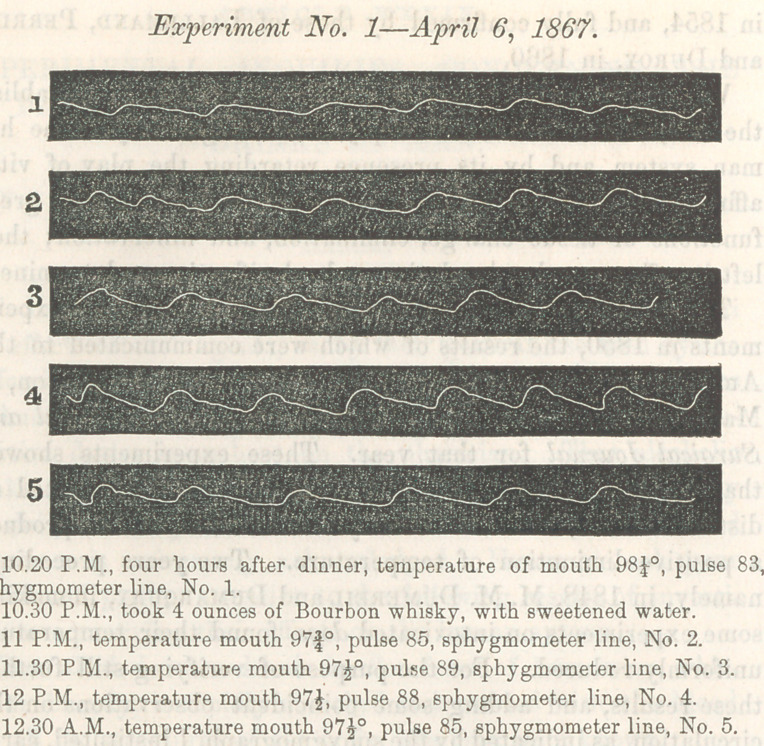


**Figure f2:**